# Peroxiredoxin 1 Controls Ovulation and Ovulated Cumulus–Oocyte Complex Activity through TLR4-Derived ERK1/2 Signaling in Mice

**DOI:** 10.3390/ijms22179437

**Published:** 2021-08-30

**Authors:** Hyo-Jin Park, Bokyung Kim, Deog-Bon Koo, Dong-Seok Lee

**Affiliations:** 1 Department of Biotechnology, College of Engineering, Daegu University, 201 Daegudae-ro, Jillyang, Gyeongsan, Gyeongbuk 38453, Korea; wh10287@naver.com (H.-J.P.); dbkoo@daegu.ac.kr (D.-B.K.); 2 Institute of Infertility, Daegu University, 201 Daegudae-ro, Jillyang, Gyeongsan, Gyeongbuk 38453, Korea; 3 BK21 FOUR KNU Creative BioResearch Group, School of Life Sciences, Kyungpook National University, Daegu 41566, Korea; mideun@knu.ac.kr

**Keywords:** cumulus–oocyte complexes (COCs), extracellular signal-regulated kinase (ERK), mice, ovulation, peroxiredoxin 1 (PRDX1), toll-like receptor4 (TLR4)

## Abstract

Peroxiredoxins (PRDXs) are expressed in the ovary and during ovulation. PRDX1 activity related to the immuno-like response during ovulation is unknown. We investigated the roles of *Prdx1* on TLR4 and ERK1/2 signaling from the ovulated cumulus–oocyte complex (COC) using *Prdx1*-knockout (K/O) and wild-type (WT) mice. Ovulated COCs were collected 12 and 16 h after pregnant mare serum gonadotropin/hCG injection. PRDX1 protein expression and COC secretion factors (*Il-6*, *Tnfaip6*, and *Ptgs2*) increased 16 h after ovulated COCs of the WT mice were obtained. We treated the ovulated COCs in mice with LPS (0.5 μg/mL) or hyaluronidase (Hya) (10 units/mL) to induce TLR4 activity. Intracellular reactive oxygen species (ROS), cumulus cell apoptosis, PRDX1, TLR4/P38/ERK1/2 protein expression, and COC secretion factors’ mRNA levels increased in LPS- and Hya-treated COCs. The ERK inhibitor (U0126) and *Prdx1* siRNA affected TLR4/ERK1/2 expression. The number and cumulus expansion of ovulated COCs by ROS were impaired in *Prdx1* K/O mice but not in WT ones. *Prdx1* gene deletion induced TLR4/P38/ERK1/2 expression and cumulus expansion genes. These results show the controlling roles of PRDX1 for TLR4/P38/ERK1/2 signaling activity in ovulated mice and the interlink of COCs with ovulation.

## 1. Introduction

Ovulation is a complex process initiated by a luteinizing hormone surge and cumulus cell (CC) expansion in cumulus–oocyte complexes (COCs) [1]. In mammals, ovulation is involved in oocyte maturation and the secretory functions of surrounding CCs. Such secretory functions are released from the surface of the ovary into the oviduct for transportation and fertilization [2]. In the CCs, a hyaluronan (HA)-rich matrix synthesized before ovulation controls the expansion of CCs, an essential process for ovulation to occur [3]. Most hyaluronan fragments are obtained from hydrolysis catalyzed by hyaluronidase (Hya) [4]. HA is a highly multifunctional molecule in many biological processes, and its level is markedly elevated during embryogenesis.

Recently, ovulation was reported to represent an inflammatory-like response through the toll-like receptor (TLR) 2/4 pathways in HA fragments induced by synthesizing an HA-rich extracellular matrix [5]. In a previous study, it was demonstrated that the expression of TLR 2/4 was found in the cumulus cells of ovulated COCs in mice [6]. The activation of TLRs can respond to specific ligands, such as lipopolysaccharide (LPS) and HA fragments, inducing inflammation and innate immune-related mechanisms [7]. During the ovulation process, CCs express innate immune cell-related proteins and cytokines, such as interleukin6 (*Il-6*) and tumor necrosis factor-alpha (TNFα). Of note, the increased expression of these innate notable anti-inflammatory/immune-modulatory factors occurs subsequently in the TNF alpha-induced protein 6 (*Tnfaip6*) and cyclooxygenase-2 (*Ptgs2*) genes [8]. Moreover, bovine granulosa cells responded acutely to LPS with rapid phosphorylation of TLR signaling components and ERK MAP kinases (P38 and ERK1/2), and increased transcriptional expression of *Il-6* [9]. In a previous study, it was described that ERK1/2 MAP kinase activation and NF-κB signaling activation were induced as a MAP kinase pathway related to immune-related mechanisms in ovulated COCs under LPS- and Hya-treated conditions. [10]. 

In female reproductive organs and germ cells, various reactive oxygen species (ROS), such as hydrogen peroxide (H_2_O_2_), are produced and utilized in some processes of the estrous cycle [11]. ROS can affect various physiological functions during ovulation and ovarian functions by changing antioxidant gene expression [12]. In addition, ROS are key signaling molecules that play an essential role in the progression of inflammatory disorders [13]. In a previous report, it was also demonstrated that the ovulatory response is commonly identified with an inflammation-like response and that the involvement of ROS production is indispensable during ovulation [14]. Nevertheless, excessive oxidative stress caused by ROS accumulation impairs hormone synthesis, oocyte maturation, ovulation, and secretory functions throughout the entire female reproductive tract [11,15].

Peroxiredoxins (PRDXs), as typical antioxidant enzymes, have an essential role in controlling intracellular functions, differentiation, and proliferation as a ROS scavenger [16]. Among the PRDX family (1–6), peroxiredoxin 1 (PRDX1) is localized in the cytosol, where it has an interaction with various signaling molecules [17,18]. Significantly, gonadotropin and granulosa cell-specific stimulation of PRDX1 are essential local regulators of follicle development [18]. In a previous study, it was shown that the *Prdx* family genes (*Prdx1*, *2*, *4*, and *6*) were detected in matured mouse COCs [19]. Moreover, PRDX1 is a ROS/MAPK-dependent inducible antioxidant that regulates NF-κB signaling and it is an antioxidant that is upregulated in a ROS/MAPK-dependent manner [20]. Although the impacts of PRDXs on ovulation have been identified, the controlling mechanisms of ROS production and TLR4-mediated signaling by PRDX1, as an antioxidant enzyme, have not been reported in the ovulation process or the ovulated COCs of mice. Moreover, studies on the underlying inflammatory mechanisms via the TLR4/ERK pathways in ovulated COCs using *Prdx1* K/O mice are still insufficient.

Controlled ROS, such as hydrogen peroxide production during ovulation, may play a fundamental role in the female reproductive tract. We thought that regulation of ROS production and oxidative stress by PRDX1 may also play a significant role in the ovulation and secretion function of the CCs of ovulated COCs in WT mice. In addition, we speculated that ROS reduction or regulation by PRDX1 would affect TLR4-derived signaling activation and secretion factors in the CCs of ovulation COCs. Therefore, in ovulated COCs, we hypothesized that ROS regulation of the antioxidant enzyme PRDX1 is involved in the secretion functions of CCs through TLR4-mediated ERK signaling. Here, we investigated ovulation-mediated TLR4/ERK protein levels after treatment with U0216 as an ERK1/2 inhibitor or *Prdx1* siRNA transfection in the ovulated COCs of WT mice to confirm the relationship between PRDX1 and TLR4/ERK signaling. Moreover, we investigated the changes in TLR4-mediated ERK signal pathways, intracellular ROS production, the number of ovulated COCs in the oviduct, and cellular apoptosis in ovulated COCs using *Prdx1* K/O mice.

## 2. Results

### 2.1. Expression of PRDX1 Protein Levels and CC Secretion Factors in Ovulated COCs of WT Mice

**Figure 1:**
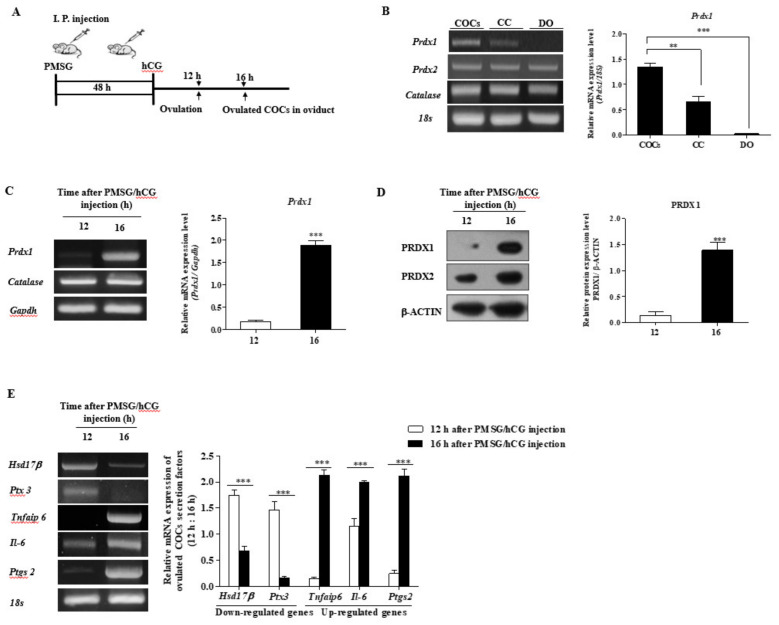
Changes in the *Prdx1* gene and secretion factor expression patterns from ovulated cumulus–oocyte complexes (COCs) at 12 and 16 h after pregnant mare serum gonadotropin (PMSG)/hCG injection. (**A**) Schematic diagram of hormone-induced superovulation in mice: 12 or 16 h after PMSG-hCG injection, ovulated COCs were obtained from the oviducts. (**B**) Expression levels of antioxidant enzymes (*Prdx1, 2,* and *Catalase*) in COCs, CC, and DOs were measured by RT-PCR analysis. (**C**) The mRNA levels of *Prdx1* and catalase as antioxidant enzymes in isolated COCs were measured by RT-PCR analysis. The mRNA levels of these genes were normalized to the *Gapdh* expression level as a control. (**D**) Protein expression levels of PRDX1 and PRDX2 in isolated COCs were measured by Western blot analysis. The levels of PRDX 1 and 2 were normalized to β-ACTIN expression level as a control. (**E**) The mRNA expression levels of ovulated COC-secreted factors (downregulated genes: *Hsd17β and Ptx3*; upregulated genes: *Tnfaip6, Il-6, and Ptgs2*) in isolated COCs were measured by RT-PCR analysis. The mRNA levels of these genes were normalized to the 18S expression level as a control. The histogram represents the values of the densitometry analysis that were obtained using Image J software. The data are representative of at least three independent experiments conducted in triplicate and shown as means ± SEM (30 COCs per group). **, *p* < 0.01; ***, *p* < 0.001 for analyses of these data via *t*-tests compared with 12 h after PMSG/hCG injection, and via Dunnett’s multiple comparison test compared with the COC sample.

In a previously published study, these two time points (12 h: immediately after ovulation; 16 h: COCs localized in the oviduct, Figure 1A) were able to extract the ovulated COCs from the oviduct. As shown in Figure 1B, we investigated the mRNA levels of *Prdx1*, -2, and catalase as antioxidant enzymes in the denuded oocytes (DO), CCs, and COCs 16 h after pregnant mare serum gonadotropin (PMSG)/hCG injection, respectively.

We also performed the RT-PCR analysis at 12 and 16 h after PMCG/hCG injection (Figure 1C) to demonstrate the mRNA expression of *Prdx1* and catalase in the ovulated COCs of WT mice. We also observed the protein levels of PRDX1 and -2 by Western blotting 12 and 16 h after PMCG/hCG injection, respectively (Figure 1D). *Prdx1* mRNA levels expressed in COCs were compared with DO or CC, and only PRDX1 protein and *Prdx1* transcription levels dramatically increased (*p* < 0.001) in ovulated COCs at 16 h after ovulation. We examined the mRNA expression of the ovulation-mediated COC secretion factors *Hsd17β, Ptx3, Tnfaip6, Il-6, and Ptgs2* (Figure 1E). As a result, the mRNA levels of upregulated genes (*Hsd17β* and *Ptx3*) for ovulation significantly decreased (*p* < 0.001), whereas the expression of downregulated genes (*Tnfaip6, Il-6,* and *Ptgs2*) for ovulation were increased (*p* < 0.001) in ovulated COCs at 16 h compared with 12 h.

These results indicate that PRDX1 expression increased dramatically in fully ovulated COCs at 16 h. Based on these results, we determined that the sample type of ovulated COCs at 16 h could be used for subsequent experiments.

### 2.2. LPS or Hya Exposure in Ovulated COCs Increased PRDX1, Cumulus Secretion Factors, and the Activity of TLR4/P38/ERK1/2 Signaling Pathways

**Figure 2:**
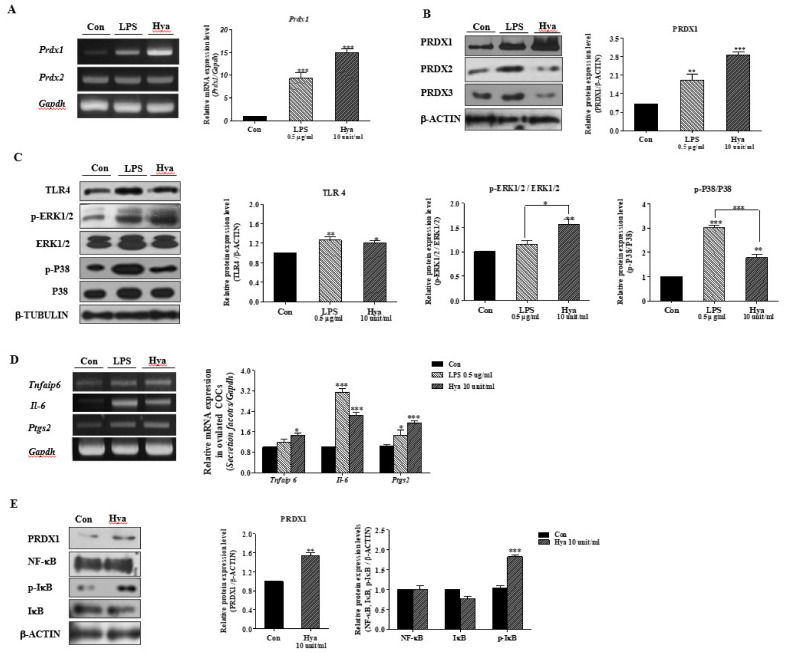
Changes in PRDX1 protein levels and TLR4-derived ERK1/2 signaling in ovulated cumulus–oocyte complexes (COCs) by LPS or Hya treatment. (**A**) The mRNA expression levels of antioxidant enzymes (*Prdx1* and *Prdx2*) and CC-secreted factors or (**D**) immune-modulatory factors (*Tnfaip6, Il-6,* and *Ptgs2*) in ovulated COCs after LPS and Hya treatment were measured by RT-PCR analysis. The mRNA levels of these genes were normalized to the *Gapdh* mRNA expression level as a control. (**B**) The protein levels of PRDXs (PRDX1, -2, and -3) and (**C,E**) TLR4-targeted signal genes (TLR4, p-ERK1/2, and ERK1/2, p-P38, P38 MAP kinase, and NF-κB signaling) in ovulated COCs after culturing with LPS or Hya were measured by Western blot analysis. The relative fold changes of these proteins were obtained by normalization to the signals for β-ACTIN and β-TUBULIN. p-ERK1/2 and p-P38 were normalized to total ERK1/2 and P38 protein. The histogram represents the values of the densitometry analysis that were obtained using Image J software. Data in the bar graph represent the means ± SEM of three independent experiments (25–30 COCs per group). *, *p* < 0.05; **, *p* < 0.01; ***, *p* < 0.001 for analysis of these data via *t*-tests and Dunnett’s multiple comparison test compared with the control group.

Lipopolysaccharide (LPS) and HA fragments produced by Hya were applied as a specific ligand for inducing TLR4 signaling pathway activation [21]. Here, we performed a Western blot analysis of TLR4, p-ERK1/2, and ERK1/2 in ovulated COCs after LPS or Hya treatment for 15 h in ovulated COCs to investigate TLR4 signaling activation. As shown in Figure 2A,B, the expression of *Prdx1* mRNA and PRDX1 protein in isolated COCs cultured with Hya (10 units/mL) increased more rapidly (*p* < 0.001) than the other groups. Next, we confirmed the activation of TLR4-mediated MAP kinase (p-ERK1/2, ERK1/2, p-P38, and P38) by LPS or Hya treatment. As expected, the protein levels of these TLR4/P38/ERK1/2 signaling factors increased significantly in ovulated COCs following LPS or Hya treatments (Figure 2C). The Hya treatment significantly increased the ERK1/2 signal compared with the LPS treatment. In contrast, the LPS treatment increased P38 activity compared with the Hya treatment. We examined the mRNA expression of TLR4-derived immune-modulatory factors or COC secretion factors *(Tnfaip6, Il-6*, and *Ptgs2*) in ovulated COCs with LPS or Hya for 15 h. Transcriptional expression of the three secretion factors from TLR4 activation in LPS- or Hya-exposed ovulated COCs increased (Figure 2D). Finally, we treated ovulated COCs with Hya for 2 h to investigate IκB/NF-κB activation as a direct TLR4 signal. The p-IκB protein level increased significantly (*p* < 0.01) in ovulated COCs, following Hya treatment for 2 h (10 units/mL) (Figure 2E).

Thus, these results are indicative that LPS- or Hya-induced TLR4 signaling pathways are similar to the inflammation response in ovulated COCs of mice by the P38/ERK1/2 MAP kinase and IκB/NF-κB signal pathways. Simultaneously, the PRDX1 expression level increased with TLR4 activation by LPS and Hya exposure in WT mouse COCs.

### 2.3. Induced ROS Production and Cellular Apoptosis in Ovulated COCs after LPS or Hya Treatment

**Figure 3:**
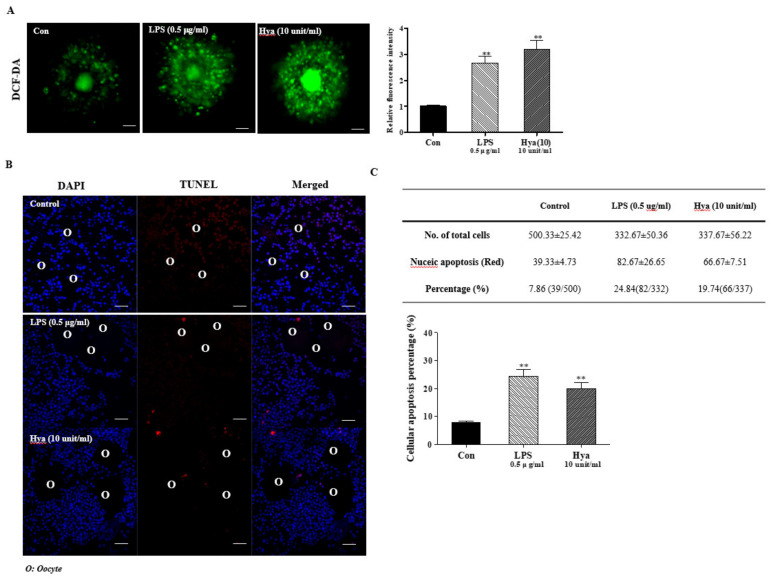
Detection of ROS production and cellular apoptosis by TUNEL staining in ovulated COCs after LPS or Hya treatment. (**A**) Detection of reactive oxygen species (ROS) production in ovulated COCs. COCs were incubated in a culture medium (M16, Sigma) with 1.0 μg/mL DCF-DA for 15 min (scale bar = 50 μm). (**B**,**C**) Detection of apoptosis in CCs of ovulated COCs by TUNEL assay analysis: Hoechst 33342 staining identifying nuclei, TUNEL staining identifying apoptotic cells, and merged photographs of Hoechst 33342- and TUNEL-stained ovulated COCs (×20; scale bar = 50 mm). The quantification of fluorescence intensity in DCF-DA- and TUNEL-stained COCs was obtained with Image J software. Data in the bar graph represent the means ± SEM of three independent experiments. **, *p* < 0.01 compared with the control.

In a previous study, it was reported that the ovulatory response was commonly identified with the involvement of ROS production [14]. In addition, excessive oxidative stress caused by ROS accumulation impaired ovulation and secretory functions [11,15,22]. Therefore, we performed DCF-DA staining to determine the production of intracellular ROS in ovulated COCs. This study was carried out to assess ROS production in TLR4 activation of in vitro-cultured ovulated COCs by LPS (0.5 μg/mL) and Hya (10 units/mL) treatment. Primarily, LPS- or Hya-treated COCs significantly increased (*p* < 0.01) the green fluorescence intensity of intracellular ROS were higher than those observed in the control group (Figure 3A). As shown in Figure 3B,C, we performed TUNEL assay staining to determine cellular apoptosis in ovulated COCs [22]. Apoptotic CCs in ovulated COCs increased significantly (*p* < 0.01) in the LPS or Hya treatment groups compared with the control. According to these results, it may be suggested that TLR4 signaling activated by LPS or Hya in ovulated COCs was induced by ROS production and the apoptosis of CCs.

### 2.4. Correlation between PRDX1 and ERK1/2 Signals in Ovulated COCs

**Figure 4:**
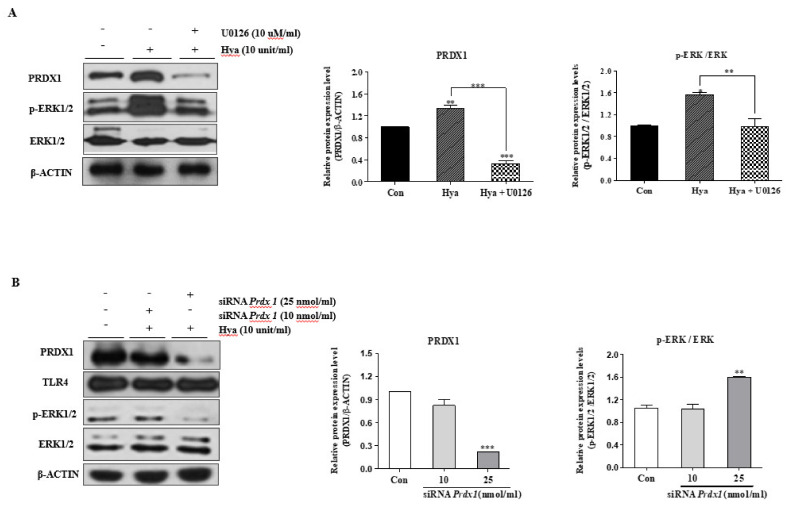
The mutual relationship of PRDX1 and ERK1/2 MAP kinase signaling in ovulated COCs using an ERK inhibitor or siRNA *Prdx1*. (**A**) Protein levels of PRDX1, ERK1/2, and p-ERK1/2 were identified by Western blot analysis in ovulated COCs after treatment with Hya or the ERK-specific inhibitor U0126. ERK1/2 inhibitor (U0126)-dependent changes in the expression of PRDX1 in COCs were cultured with Hya (10 units/mL) treatment. (**B**) siRNA *Prdx1*-dependent changes in the activation of TLR4-mediated ERK1/2 pathways in ovulated COCs were cultured with Hya (10 units/mL) treatment. Ovulated COCs were cultured with Hya for 15 min for ERK1/2 activation; after 2 h, they were treated with siRNA *Prdx1* (10 and 25 nmol/mL). We performed a Western blot analysis to confirm the protein levels of MAPK signaling genes (p-ERK1/2 and ERK1/2), PRDX1, and TLR4 in ovulated COCs after with *Prdx1* siRNA treatment. These gene protein levels were normalized to β-ACTIN expression as a control. The p-ERK1/2 was normalized to total ERK1/2. Histogram values of the densitometry analysis were obtained using Image J software. Bar graph data represent the least-squares means ± SEM (30 COCs per group) of three independent experiments. *, *p* < 0.05; **, *p* < 0.01; ***, *p* < 0.001 for analysis of these data via Dunnett’s multiple comparison test compared with the control group.

Figure 2 indicates that PRDX1 and TLR4-targeted ERK1/2 signals are induced by LPS or Hya exposure in ovulated COCs. Therefore, we treated the ERK inhibitor (U016; 10 μM/mL) or siRNA *Prdx1* (10 or 25 nmol/mL) to examine the relationship between PRDX1 and TLR4-targeted ERK1/2 signals in the ovulated COCs of WT mice. The PRDX1 protein level decreased significantly (*p* < 0.001) in ovulated COCs cultured with an ERK1/2-specific inhibitor after Hya stimulation (Figure 4A). As shown in Figure 4A, the PRDX1 protein expression levels increased significantly (*p* < 0.01) and depended only on the Hya treatment. On the contrary, PRDX1 expression was reduced considerably (*p* < 0.001) by the ERK1/2 inhibitor (U0126) in Hya-exposed ovulated COCs.

We then investigated the changes in TLR4-derived ERK1/2 signaling by Western blot analysis in COCs after siRNA *Prdx1* transfection to determine ERK1/2 MAP kinase signal activation of the suppressive effects of *Prdx1* gene expression by siRNA *Prdx1* in ovulated COCs (Figure 4B). As a result, COCs transfected with 25 nmol/mL *Prdx1* siRNA significantly increased (*p* < 0.001) ERK1/2 activity. These results are indicative of a possible correlation between PRDX1 and TLR4-mediated ERK1/2 MAP kinase in ovulated COCs in mice.

### 2.5. Obstruction of (Decreasing) Ovulated COC Secretion Function and Expansion via TLR4-Mediated p38 and ERK1/2 Signaling in Prdx1 Knockout (K/O) Mice

**Figure 5:**
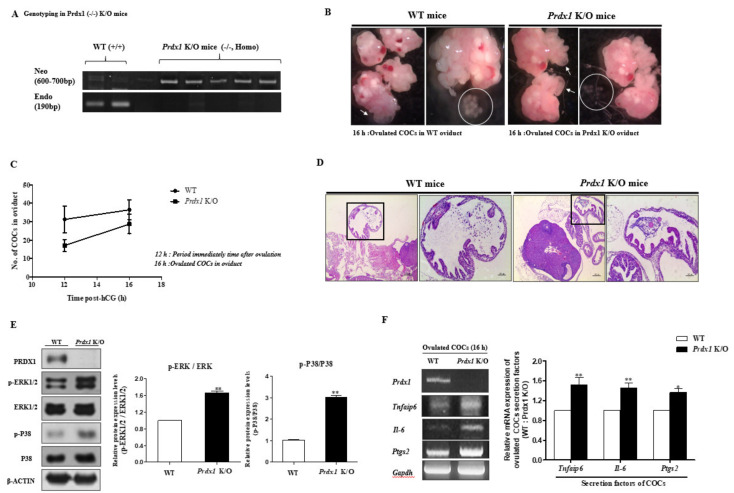
Effects of *Prdx1* defection on the number of ovulated COCs, COC-secreted cytokines, and the ERK1/2 signaling pathway in *Prdx1* K/O mice. (**A**) We performed genotyping of the *Prdx1* gene by PCR in the tails of *Prdx1* K/O mice and WT mice. (**B**) Microscope images showing the number of ovulated COCs taken directly from the oviduct in *Prdx1* K/O mice compared with WT mice. (**C**) Quantification and graph of the reduced number of ovulated COCs in *Prdx1* K/O mice. (**D**) Ovulated COC number and CC expansion in the oviduct of *Prdx1* K/O mice were observed by hematoxylin and eosin (H&E) staining. (**E**) TLR4 signal-mediated ERK1/2 MAP kinase pathway in the ovulated COCs of *Prdx1* K/O mice analyzed by Western blotting for the TLR4-derived MAP kinase signaling markers (p-P38, P38, p-ERK1/2, and ERK1/2) and PRDX1 protein expression in *Prdx1* K/O mice. These protein levels were normalized to β-ACTIN expression as a control. The p-ERK was normalized to total ERK1/2 protein. (**F**) Changes in the mRNA levels of *Prdx1* and COC secretion factors (*Tnfaip6, Il-6,* and *Ptgs2*) were investigated in ovulated COCs of *Prdx1* K/O mice by RT-PCR analysis. The levels of these genes were normalized to the *Gapdh* mRNA expression level as a control. Histogram values of the densitometry analysis were obtained using ImageJ software. Bar graph data represent the least-squares means ± SEM of three independent experiments. (*, *p* < 0.05, **, *p* < 0.01 compared with WT mice).

The genotypes of *Prdx1* K/O mice were confirmed by PCR analysis, and a band of the Neo gene was represented in 600–700 bp versus 190 bp in the WT (Figure 5A). We observed that the *Prdx1* K/O (−/−) mice had some characteristic differences in ovulation and the number of ovulated COCs compared with female WT mice shown by H&E staining and microscope image data (Figure 5B–D). As shown in Figure 5B,C, the COCs collected from the oviducts of *Prdx1* K/O mice 16 h after a PMSG/hCG injection were an indication that ovulation still occurred, but the formation or number of COCs showed several abnormalities. In WT mice, the CCs of ovulated COCs were well structured (Figure 5D, left panel), with CCs radiating from a central oocyte. On the contrary, we observed an abnormal pattern of CC expansion in ovulated COCs in the oviduct of *Prdx1* K/O mice (Figure 5D, right panel). In addition, we investigated the p-ERK1/2, ERK1/2, p-P38, and P38 protein levels using Western blotting analysis in the COCs of *Prdx1* K/O mice to determine the changes in TLR4-derived p38 and ERK1/2 signaling induced by *Prdx1* deficiency in ovulated COCs. As shown in Figure 5E, the protein expression levels of p-ERK1/2 and p-P38 increased significantly (*p* < 0.01) in the ovulated COCs of *Prdx1* K/O mice. These observations indicate that the p38 and ERK1/2 signaling pathways increased the *in vivo* levels of ovulated COCs in *Prdx1* K/O compared with WT mice. The mRNA expression of *Tnfaip6, Il-6,* and *Ptgs2* increased in the ovulated COCs of *Prdx1* K/O compared with WT mice 16 h after PMSG/hCG injection (Figure 5F).

**Figure 6:**
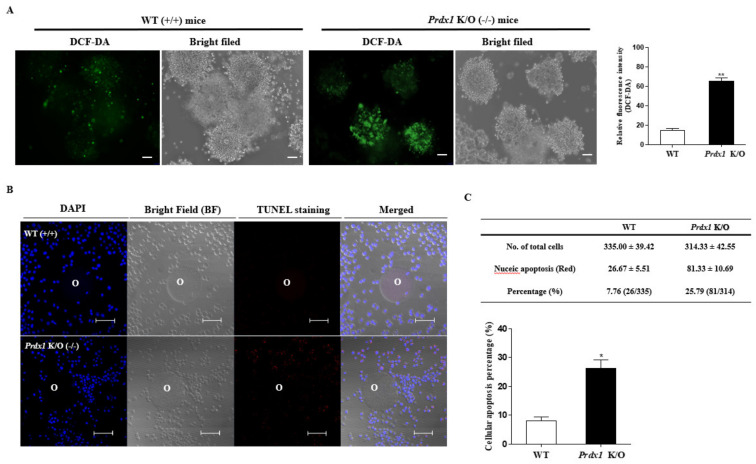
ROS production and cellular apoptosis according to *Prdx1* gene deficiency in the ovulated COCs of *Prdx1* K/O mice. (**A**) Investigation of intracellular ROS production in ovulated COCs by *Prdx1* deletion. COCs of *Prdx1* K/O and WT mice were incubated in a culture medium (M16, Sigma) with 1.0 μg/mL DCF-DA for 15 min (scale bar = 50 μm). (**B**,**C**) Detection of cellular apoptosis in CCs of ovulated COCs in *Prdx1* K/O mice, with Hoechst 33342 staining identifying nuclei, TUNEL staining identifying apoptotic cells, and a merged photograph of Hoechst 33342 and TUNEL-stained ovulated COCs (20×; scale bar = 50 mm) of *Prdx1* K/O mice. The quantification of fluorescence intensity in DCF-DA- and TUNEL-stained COCs was obtained using the Image J program. Data in the bar graph represent the means ± SD of three independent experiments. *, *p* < 0.05; **, *p* < 0.01 compared with WT mice.

We performed DCF-DA staining in the ovulated COCs of *Prdx1* K/O mice to analyze the changes in ROS production induced by *Prdx1* gene deletion. The ROS levels of ovulated COCs in *Prdx1* K/O mice were higher than in WT mice (Figure 6A). These results show that it may be that the ovulated COCs have a high level of ROS due to a decline in *Prdx1* as an antioxidant enzyme in the ovulated COCs of *Prdx1* K/O mice. As shown in Figure 6B, the apoptotic cells of ovulated COCs rapidly increased (*p* < 0.01) with antioxidant *Prdx1* deletion in *Prdx1* K/O mice. These results are an indication that the formation of complete COCs was reduced, and TLR4-derived ERK1/2 signal-induced secretion factor expression, ROS production, and cellular apoptosis increased significantly with a deficiency of the *Prdx1* gene as an antioxidant enzyme in ovulated COCs of *Prdx1* K/O mice.

## 3. Discussion

In this study, we demonstrated that the interrelation between the PRDX1 protein as a ROS scavenger and the ovulatory process is associated with cumulus secretion factors (*Tnfaip6*, *Il-6*, and *Ptgs2*) from ovulated COCs through TLR4-mediated p38 and ERK1/2 signaling. In addition, these results are indicative that *Prdx1* K/O female mice reduced the number of ovulated COCs by increasing intracellular ROS production and cellular apoptosis.

Ovulation is a complex process that involves marked changes in follicular cell functions [23]. In addition, ovulation is known as an immune-like response caused by TLR4 activation and secreted cytokines. The function and expression of innate immune cell-related cytokines or secreted factors (*Il-6*, *Tnfaip6*, and *Ptgs2*) in non-immune cells named COCs in the ovary have been studied [5,24]. As shown in Figure 1, the mRNA expression of innate immune cell-related cytokines or target genes (*Tnfaip6* and *Il-6*), involved in the inflammation response [25], increased rapidly in ovulated COCs 16 h after PMSG/hCG injection.

Primarily, CCs produce and are surrounded by an HA-rich extracellular matrix, and HA fragments have been shown to activate TLR2 and TLR4 [25]. In a previous study, it was demonstrated that HA fragments from the Hya generated during matrix degradation might act as an endogenous ligand for TLR2 and TLR4 occurring in the CCs of ovulated COCs. TLR4 is activated by bacterial lipopolysaccharide (LPS) or, specifically, the enzyme of hyaluronidase (Hya) [26]. Therefore, we used LPS treatment and culture with Hya to induce activation of TLR4 in ovulated COCs. Notably, the results presented here are indicative that exposure of ovulated COCs to HA fragments was induced by the phosphorylation of ERK1/2 MAP kinase and NF-κB, and the expression of secretion genes (*Tnfaip6*, *Il-6*, and *Ptgs2*) from THE CCs of ovulated COCs (Figure 2). These responses were similar when ovulated COCs were exposed to LPS, a known ligand of TLR4. The HA fragments could serve as ligands for TLR4-derived MAP kinase activity, similar to cytokine secretion in THE CCs of ovulated COCs.

ROS production and oxidative stress are known to contribute to female ovarian function and reproductive processes such as ovulation [11]. ROS generation is dynamically modulated as a reason for and as a result of many cellular events, including the immune response, cell apoptosis, and stress [27]. However, the roles of many antioxidant enzymes have been reported for PRDXs (PRDX2, -4, and -6) and catalase in female reproduction processes [28,29,30]. The direct effects of PRDX1 on the cytokine secretory functions of ovulated COCs through TLR4/ERK MAP kinase signaling have not been reported. In our results, it was shown that *Prdx1* mRNA levels increased significantly in the COC sample type compared with CC and DO (Figure 1B). As shown in Supplementary Figure S2, the mRNA levels of *Prdx2*, *-4*, *-5*, and *-6* were not changed by *Prdx1* gene deficiency in the ovulated COCs of *Prdx1* K/O mice. Therefore, these findings suggest a close relationship between *Prdx1* and ovulated COCs during the ovulation process in mice.

In addition, the TLR-mediated signaling pathways have a reasonably close relationship with intracellular ROS production [31]. As shown in Figure 3, under activation of TLR4 in ovulated COCs by Hya or LPS, ROS production and cellular apoptotic cells were significantly increased. PRDX1 has previously been demonstrated to be a versatile molecule regulating cell growth, differentiation, apoptosis, TLR signal activation and TLR-derived ROS production [32], ERK12/ MAP kinase [33], and NF-κB signal uptake [34]. Generally, increased ROS production in immune cells leads to the activation of ERK and MAPK signaling by TLR activation. However, the mechanisms by which the antioxidant enzymes PRDX1 can activate TLR4/ERK MAP kinases are unclear in the ovulatory response. In the present study, we identified the connection mechanism between PRDX1 and ERK1/2 signaling in ovulated COCs using siRNA *Prdx1* and the specific ERK1/2 inhibitor U0126 (Figure 4). We showed that siRNA *Prdx1*-treated COCs had significantly increased expression levels of ERK1/2 signals but no change in TLR4 protein levels. Interestingly, inhibiting the activation of ERK1/2 signaling rapidly decreased the protein levels of PRDX1 in ovulated COCs. These findings are indicative that PRDX1 may be associated with the activation of ERK1/2 signaling pathway in the activated cumulus of ovulated COCs. Here, we provide the first evidence that PRDX1 can play a cytokine secretion and CC functional role in the ovulated COCs or ovulatory process through TLR4-derived ERK1/2 activation.

Oxidative stress, such as hydrogen peroxide (H_2_O_2_), influences the ovulation process [11]. PRDX1 is upregulated following exposure to oxidative stress [18]. Antioxidant PRDX1 promotes MAP kinase [33] and TLR4/NF-κB activation in inflammation [35]. Thus, increased ERK1/2 signaling pathways in *Prdx1* K/O mice may result from the absence of *Prdx1*-dependent antioxidative surveillance in ovulated COCs. An increase in ROS levels and apoptotic cells in *Prdx1* K/O mice may indicate overexpression of the MAP kinase signal and secretion factor activity in ovulated COCs as a reason for *Prdx1* deficiency.

In particular, as shown in Figure 5 and Figure 6, these results are demonstrations that the *Prdx1* deficiency in ovulated COCs of *Prdx1* K/O mice was induced by the incomplete formation of COCs, the overexpression of cumulus-related secretion factors, high levels of ROS production, and an increase in TLR4-derived p38 and ERK1/2 activation. Moreover, apoptotic cells and intracellular ROS levels increased significantly in the ovulated COCs of *Prdx1* K/O mice. These results may be evidence of the role of PRDX1 as a ROS scavenger for TLR activation in ovulation-related processes or ovulated COC formation. In a previous study, it was demonstrated that follicle-stimulating hormones, such as progesterone and estrogen in granulosa cells, which might play important roles during the follicle maturation process, are regulated by the FSH-activated p38 MAPK signaling pathway [36]. An increase in phosphorylated p38-MAPK protein expression during oocyte maturation has also been reported [37]. Despite the possibility of FSH-induced P38 MAP kinase activity in oocyte and steroid hormone production during the follicle maturation process, in our findings, we showed that TRL4 targets other downstream substrates, ERK1/2, of the PRDX1 that protects the antioxidant response to ROS (Figure 4). Ovulation is dependent on proper ERK1/2 signaling, as several genes in the GCs of follicular fluid are required for the ovulatory process and are dysregulated in the absence of ERK1/2 MAP kinase [38]. Recently, ROS was shown to have an important role in the normal functioning of the reproductive system and in the pathogenesis of infertility in females. Problems with secretion factors and various antioxidant enzymes from ovulated COCs display a severe defect in fertility capacity [12,39]. Possibly, the control of ROS production by PRDX1 has a fundamental role during ovulation in the female reproductive system.

**Figure 7:**
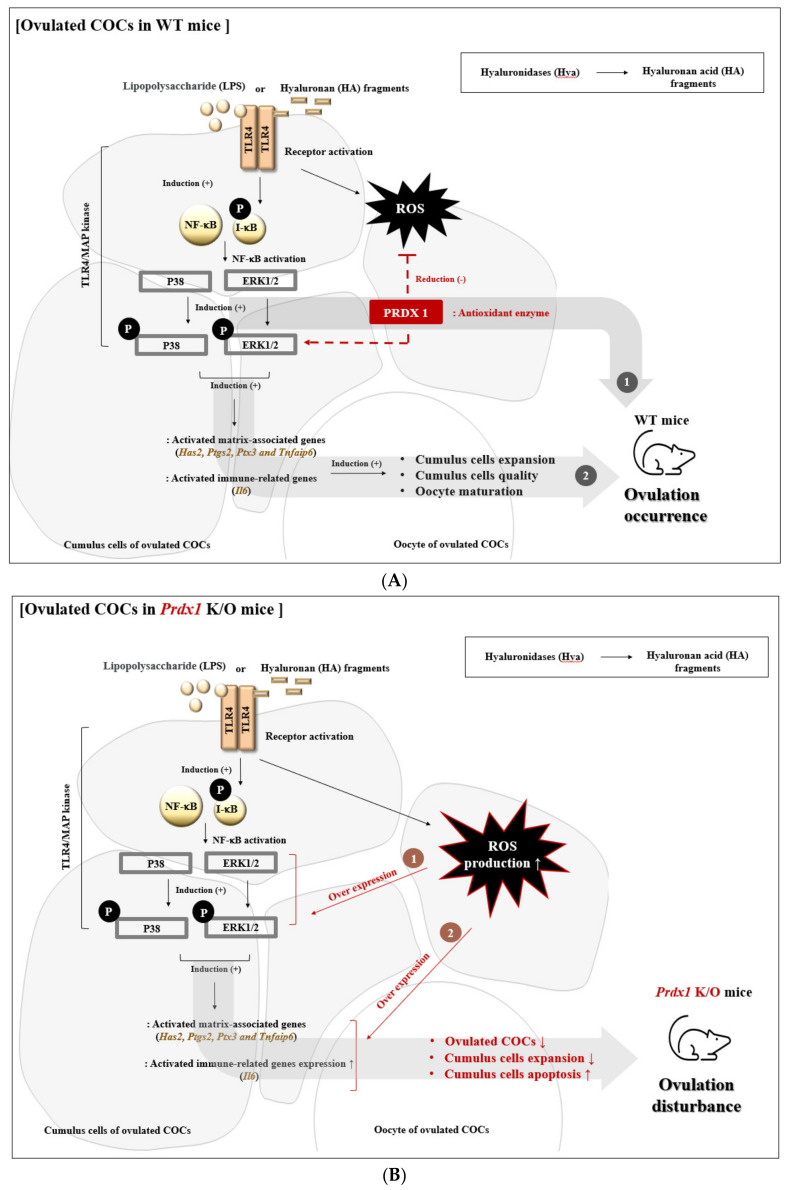
(**A**) WT mice: Roles of PRDX1 in cytokine secretion function and the ovulatory process associated with the TLR4-derived ERK1/2 MAP kinase activity in the ovulated COCs of mice, showing the effect of PRDX1 on the secretory functions (*Tnfaip6, Il-6*, and *Ptgs2)* of cumulus cells, leading to ovulation by activation of TLR4-mediated ERK signaling in ovulated COCs. (**B**) *Prdx1* K/O mice: In the present study, these observations demonstrated novel evidence of the effects of *Prdx1*, as a directly upregulated response to ERK1/2 signaling for regulating ROS production, on the secretory functions of cumulus cells by TLR4 pathways in ovulated COCs from *Prdx1* K/O mice.

In summary, our findings indicate that Prdx1 is essential to preserve the functional integrity of ovulation and ovulated COC-linked oxidative damage through TLR4/ERK1/2 signal activation in WT mice (Figure 7A). In the case of WT mice, the ROS production from TLR4/ERK1/2 pathway activity was regulated by the role of antioxidant enzymes including Prdx1; therefore, we speculated that these mice showed normal ovulation and numbers of ovulated oocytes. Moreover, the *Prdx1* gene deficiency-induced excessive ROS generation decreased the number of ovulated COCs, and the oversecretion of CC expansion factors via overexpression of TLR4 mediated ERK signaling activation in ovulated COCs from *Prdx1* K/O mice (Figure 7B). Therefore, in the present study, we have provided the first evidence of the protective effects of the *Prdx1* gene in the secretory functions of CCs and the ovulatory response associated with the TLR4-derived ERK1/2 signaling pathways in the ovulated COCs of *Prdx1* K/O mice. In other words, as an upregulator of TLR4/ERK1/2 signaling that can be regulated the cytokine secretion of cumulus cells from PRDX1, PRDX1 is judged to prevent excessive ROS production and an ERK1/2 overexpression-induced decrease in ovulated oocytes number during the ovulation process of WT mice.

## 4. Materials and Methods

### 4.1. Chemicals

Unless otherwise stated, all chemicals and reagents used in this study were purchased from Sigma-Aldrich (St. Louis, MO, USA).

### 4.2. Animals

Wild-type Female C57/B6J mice (8–10 weeks old) were purchased from Hyochang Bioscience (Daegu, South Korea), and *Prdx1*-deficient (*Prdx1* –/–, knockout (K/O)) female mice (8–10 weeks old) were maintained following the institutional guidelines of the Institutional Animal Care and Use Committee of the Korean Research Institute of Bioscience and Biotechnology (KRIBB, Daejeon, South Korea, permit number: KRIBB-AEC-17092). According to the KRIBB guidelines for the care and use of laboratory animals, the mice were housed individually in standard cages and maintained at 22 °C ± 2 °C in a room with a 12-h light/dark cycle. Fresh food and water were provided at all times. All procedures were performed under anesthesia by inhalation of isoflurane, and all efforts were made to minimize suffering.

### 4.3. Isolation and Culture of Ovulated COCs

Mice received intraperitoneal injections of 5 IU of PMSG (Sigma, St. Louis, MO, USA) followed by 5 IU of human chorionic gonadotropin (hCG; Sigma), 48 h apart. Nine mice from each strain were sacrificed (3 mice × 3 replicates) 12 and 16 h after hCG injection. Ovaries and oviducts from each mouse were removed and placed in an M-2 collection medium or TL-HEPES in 1 well of a 4-well culture plate. The oviducts were flushed with M-2 medium, and the number of oocytes from each mouse was counted under a binocular Nikon dissecting microscope. Both mouse strains began ovulating 12 h after hCG administration. Ovum counts peaked 12 h after PMSG/hCG injection in C57BL/6 mice, and localization in the ampulla of the oviduct occurred 16 h after injection in C57BL/6 mice [40] (Supplementary Figure S1: graphical description of these methods). Ovulated COCs were recovered from oviducts, and COCs (30 from each) were cultured in separate wells of a Falcon 4-well plate (Becton Dickinson, Franklin Lakes, NJ) in 500 μL of the defined medium, with LPS (0.5 μg/mL) or Hya (10 units/mL) treatment for 15 min. Next, we collected the COCs. The ovulated COCs were cultured with LPS (0.5 μg/mL) or Hya (10 unit/mL) for 15 min to confirm ERK1/2 MAP kinase activation, or for 2 h to investigate the activation of IκB and NF-κB signaling. These experimental methods have been described previously [10].

### 4.4. Measurement of Intracellular ROS Using DCF-DA Staining

COCs treated with LPS (0.5 μg/mL) or Hya (10 units/mL) were harvested by trypsinization (0.05% trypsin-EDTA, Welgene, Daegu, Korea). Treated COCs were washed with phosphate-buffered saline–polyvinylpyrrolidone (PBS-PVP, pH7.4, PVP was diluted in 1 M PBS) and COCs were incubated for 15 min at 37.5 °C with 2.5 μM CM-H2 DCF-DA (Invitrogen, CA, USA) to measure ROS levels. DCF-DA was used for detecting H_2_O_2_ [41]. After that, the COCs were then washed twice with PBS-PVP and observed with an Olympus BX51 microscope (Olympus, Japan) under a bright field. Images were acquired with an Olympus DP 70 camera (Olympus).

### 4.5. Assessment of Apoptosis in CCs of Ovulated COCs

A portion of the COCs treated with/without LPS (0.5 μg/mL) or Hya (10 unit/mL) were fixed in 4% paraformaldehyde (PFA) in PBS for the detection of apoptosis by terminal deoxynucleotidyl transferase-mediated dUDP nick-end labeling (TUNEL) using a fluorescein isothiocyanate-conjugated in situ cell death detection kit (Roche, Penzberg, Germany) according to the manufacturer’s instructions. Briefly, the COCs were fixed in 4% PFA containing 30 μg/mL Hoechst for 20 min, then permeabilized using 1% Tripton for 5 min and incubated in the TUNEL reagent for 1 h at 37 °C. The stained COCs were then washed, mounted, and examined with an LSM-710 confocal microscope (Carl Zeiss, Germany).

### 4.6. RNA Extraction and Reverse Transcription (RT)-PCR

Total RNA was isolated from ovulated COCs of mice after PMSG-hCG treatment according to the manufacturer’s instructions using Trizol reagent (Invitrogen, Carlsbad, CA, USA). RNA concentration and purity were measured with a NanoDrop spectrophotometer (ACTGene, Piscataway, NJ, USA). Subsequently, 1 μg/μL of total RNA and AccuPower RT-PCR Premix (Bioneer Inc., Daejeon, South Korea) were used to synthesize cDNA. Primers specific for the sequences of interest (Table 1 and Table 2) were designed using the NCBI database. The PCR was carried out at 95 °C for 5 min, followed by 30 cycles comprising the following steps: 95 °C for 30 s, 55–60 °C for 30 s, 72 °C for 30 s, and 72 °C for 5 min. The PCR products were separated by electrophoresis on a 2% agarose gel, stained with ethidium bromide (EtBr), and photographed under UV illumination. Band intensities were quantified using ImageJ software (National Institutes of Health, MD).

### 4.7. Protein Extraction and Western Blot Assays

Ovulated COC lysates were prepared in an ice-cold PRO-PREP protein lysis buffer (iNtRON, Daejeon, South Korea). The protein concentration of each sample was estimated using a Bradford dye-binding assay. Aliquots of the proteins (30 μg) were separated with SDS-PAGE in 12% gels. After electrophoresis, the separated proteins were transferred onto nitrocellulose membranes (Pall Corporation, Port Washington, NY). After blocking with 5% non-fat milk in Tris-buffered saline with 0.1% Tween 20 at 4 °C with shaking, the membranes were incubated with the following antibodies: anti-TLR4 (Santa Cruz), anti-ERK1/2 (cell signaling), anti-p-ERK1/2 (cell signaling), anti-p-P38 (cell signaling), anti-P38 (cell signaling), anti-p-P38 (cell signaling), anti-PRDX1 (Abfrontia), anti-PRDX2 (Abfrontia), anti-PRDX3 (Abfrontia), anti-β-ACTIN (Santa Cruz), anti-NF-κB (Santa Cruz), anti-IκB (Santa Cruz), anti-p-IκB (Santa Cruz), and anti-β-tubulin (cell signaling). The membranes were then incubated with a secondary antibody, HRE-conjugated anti-goat/mouse/rabbit IgG (Thermo, Rockford, IL). Next, the membranes were washed with a TBST buffer. Antibody binding was detected using an ECL kit (Advansta Corp., Melno Park, CA). For signal quantification, the bands were scanned using ImageJ software.

### 4.8. Transfection of SiRNA for the Mouse Prdx1 Gene

Two predesigned potential *Prdx1* siRNAs were chemically synthesized by Bioneers (Seoul, Korea) and deprotected, and annealed CCs of ovulated COCs were transfected with *Prdx1* siRNA (10 and 25 nM) using Lipofectamine RNAiMAX, according to the manufacturer’s instructions. Treatment with Hya (10 unit/mL) was performed 24 h after siRNA transfection. Sequences of *Prdx1* and non-specific control siRNAs were as follows: mouse *Prdx1* siRNA, #1: GUA UAU GUG AGG CUA GUA A (sense), #2: UUA CUA GCC UCA CAU CUC C (antisense); non-specific control siRNA: CCU ACG CCA CCA CUU UGG U (sense).

### 4.9. Statistical Analysis

All experiments were repeated at least 3 times, and the percentage data obtained in this study were presented as the mean ± standard error of the mean (SEM). These data were subjected to a one-way analysis of variance, followed by Dunnett’s and Bonferroni’s multiple comparison test. Statistical analysis was performed using paired Student’s *t*-test. Data obtained from ovulated COCs of WT and *Prdx1* K/O mice were compared using the unpaired Student’s *t*-test. All calculations were performed using the GraphPad Prism 5.0 software package (GraphPad Software, San Diego, CA, USA). Histogram values of the densitometry analysis were obtained using Image J software. Differences were considered significant at *, *p* < 0.05, **, *p* < 0.01, and ***, *p* < 0.001.

## Figures and Tables

**Table 1:** Primer sequence for reverse transcription PCR in ovulated COCs related genes.

Target	Primer	Sequence Reported 5’-3’	Tm °C	Amplicon Length (bp)
***Hsd17βB3*** ***(NM_008291.3)***	Sense	ATGGAGTCAAGGAGGAAAGGC	61	556
	Antisense	GGCTGTAAAGAGGCCAGGG		
***Ptx3*** ***(NM_008987)***	Sense	GGACAACGAAATAGACAATGGACTT	55	109
	Antisense	CGAGTTCTCCAGCATGATGAAC		
***Il-6*** ***(NM_001314054.1)***	Sense	AGTTGCCTTCTTGGGACTGA	57	223
	Antisense	TTCTGCAAGTGCATCATCGT		
***Ptgs2*** ***(NM_011198.3)***	Sense	TGTACAAGCAGTGGCAAAGG	53	433
	Antisense	CAATGTGCAAGATCCACAGC		
***Tnfaip6*** ***(NM_009398)***	Sense	TTCCATGTCTGTGCTGCTGGATGG	64	330
	Antisense	TTTGACCTTGAACATGATCCAGGCT		

*17βHSD*; 17β-hydroxysteroid-dehydrogenase type 3, *Ptx3*; Pentraxin 3, Il-6; Interleukin 6, *Ptgs2;* prostaglandin-endoperoxide synthase 2, *Tnfaip6;* tumor necrosis factor alpha induced protein, Tm; melting temperature.

**Table 2:** Primer sequence for reverse transcription PCR.

Target	Primer	Sequence Reported 5’-3’	Tm °C	Amplicon Length (bp)
***Prdx1*** ***(NM_011034)***	Sense	CACCCAAGAAACAAGGAGGA	53.5	343
	Antisense	TGGTCCAGTGCTCACTTCTG		
***Prdx2*** ***(NM_011563)***	Sense	AGGACTTCCGAAAGCTAGGC	54.1	387
	Antisense	CCTTGCTGTCATCCACATTG		
***Prdx3*** ***(NM_007452)***	Sense	CTCTGCCCAAGGAAAGTCAG	53.3	463
	Antisense	GACACTCAGGTGCTTGACGA		
***Prdx4*** ***(NM_016764)***	Sense	TCTCCAAGCCAGCACCTTAT	53.2	429
	Antisense	ATCTTCCGACAGGAAGGTCA		
***Prdx5*** ***(NM_012021)***	Sense	GGCATTTACACCTGGCTGTT	53.5	297
	Antisense	AGTGCCTTCACTATGCCGTT		
***Prdx6*** ***(NM_007453.4)***	Sense	CCTGGAGCAAGGACATCAAT	53.5	326
	Antisense	TACCATCACGCTCTCTCCCT		
***Catalase*** ***(NM_009804)***	Sense	GCGTCCAGTGCGCTGTAGA	59.2	199
	Antisense	TCAGGGTGGACGTCAGTGAA		
***18s*** ***(NR_003278.3)***	Sense	GTAACCCGTTGAACCCCATT	57	151
	Antisense	CCATCCAATCGGTAGTAGCG		
***Gapdh*** ***(BC145810.1)***	Sense	ACCACAGTCCATGCCATCAC	55	452
	Antisense	TCCACCACCCTGTTGCTGTA		

*Prdx1*: Peroxiredoxin 1, *Prdx2*: Peroxiredoxin 2, *Prdx3*: Peroxiredoxin 3, *Prdx4*: Peroxiredoxin 4, *Prdx5*: Peroxiredoxin 5, *Prdx6*: Peroxiredoxin 6, Tm; melting temperature.

## Data Availability

Data is contained within the article or Supplementary Materials.

## Supplementary Materials

The following are available online at https://www.mdpi.com/article/10.3390/ijms22179437/s1, Figure S1. Collection of ovulated COCs in mouse oviduct after 16 h PMSG/hCG injection. Figure S2. Changes in mRNA levels of Prdxs family genes (Prdx 1,2,4,5 and 6) in ovulated COCs in WT and Prdx1 K/O mice.
