# Supplementary material for: Peroxiredoxin 1 Controls Ovulation and Ovulated Cumulus–Oocyte Complex Activity through TLR4-Derived ERK1/2 Signaling in Mice
Source: Int J Mol Sci. 2021 Aug 30;22(17):9437. doi: 10.3390/ijms22179437 (PMC8430854; doi:10.3390/ijms22179437)
Supplement: Supplementary file 1 [file ijms-22-09437-s001.zip › IJMS_Supple. Fig.2 COCs_Prdx1-6 20210806.pptx]

## Slide 1
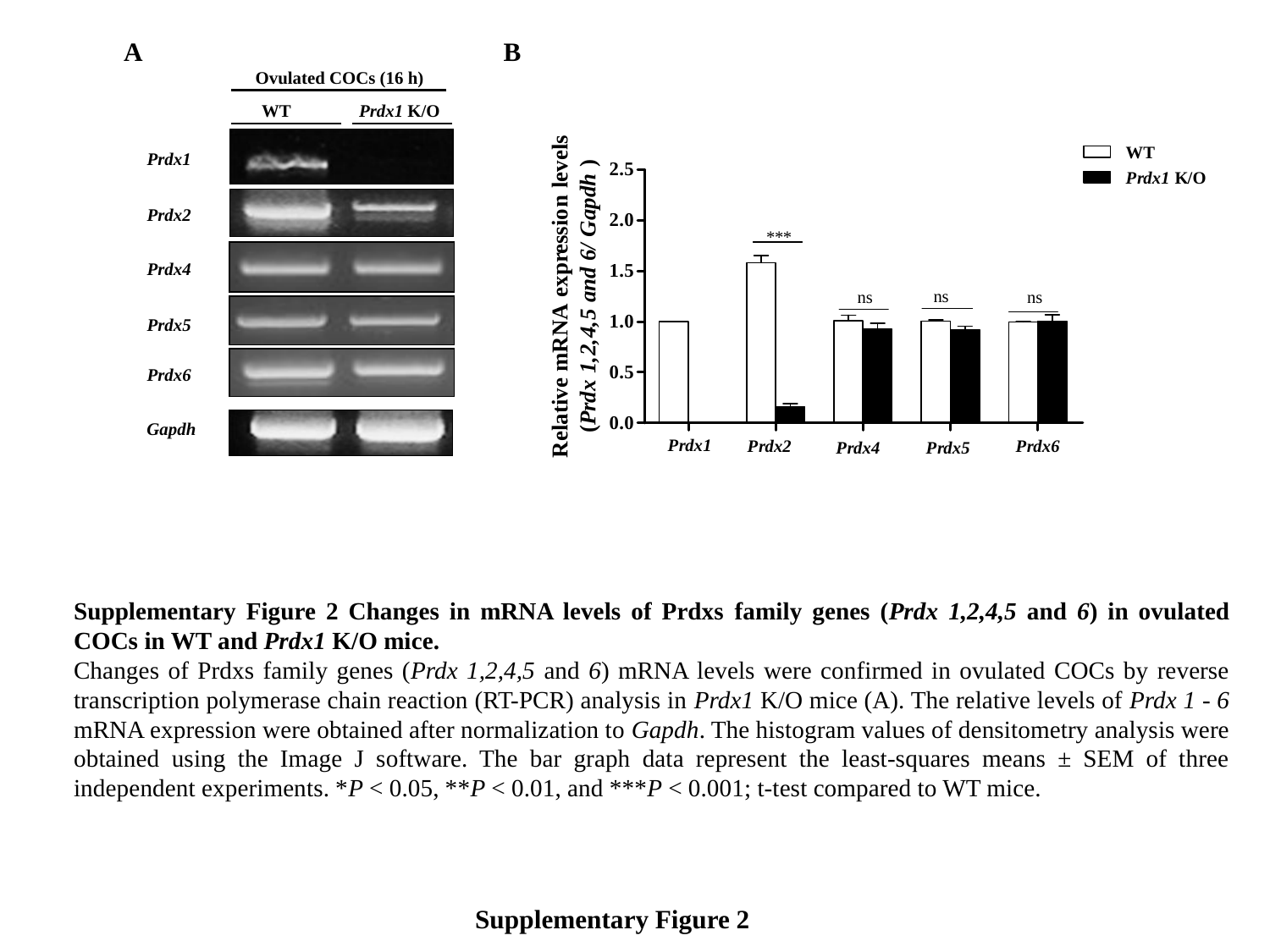

B
A
Ovulated COCs (16 h)
WT
Prdx1 K/O
Prdx1
Prdx2
Prdx4
Prdx5
Prdx6
Gapdh
Supplementary Figure 2 Changes in mRNA levels of Prdxs family genes (Prdx 1,2,4,5 and 6) in ovulated COCs in WT and Prdx1 K/O mice.
Changes of Prdxs family genes (Prdx 1,2,4,5 and 6) mRNA levels were confirmed in ovulated COCs by reverse transcription polymerase chain reaction (RT-PCR) analysis in Prdx1 K/O mice (A). The relative levels of Prdx 1 - 6 mRNA expression were obtained after normalization to Gapdh. The histogram values of densitometry analysis were obtained using the Image J software. The bar graph data represent the least-squares means ± SEM of three independent experiments. *P < 0.05, **P < 0.01, and ***P < 0.001; t-test compared to WT mice.
Supplementary Figure 2
