# Supplementary material for: Peroxiredoxin 1 Controls Ovulation and Ovulated Cumulus–Oocyte Complex Activity through TLR4-Derived ERK1/2 Signaling in Mice
Source: Int J Mol Sci. 2021 Aug 30;22(17):9437. doi: 10.3390/ijms22179437 (PMC8430854; doi:10.3390/ijms22179437)
Supplement: Supplementary file 1 [file ijms-22-09437-s001.zip › IJMS_Supple. Fig.1 Collection _COCs (M&M) 20210806.pptx]

## Slide 1
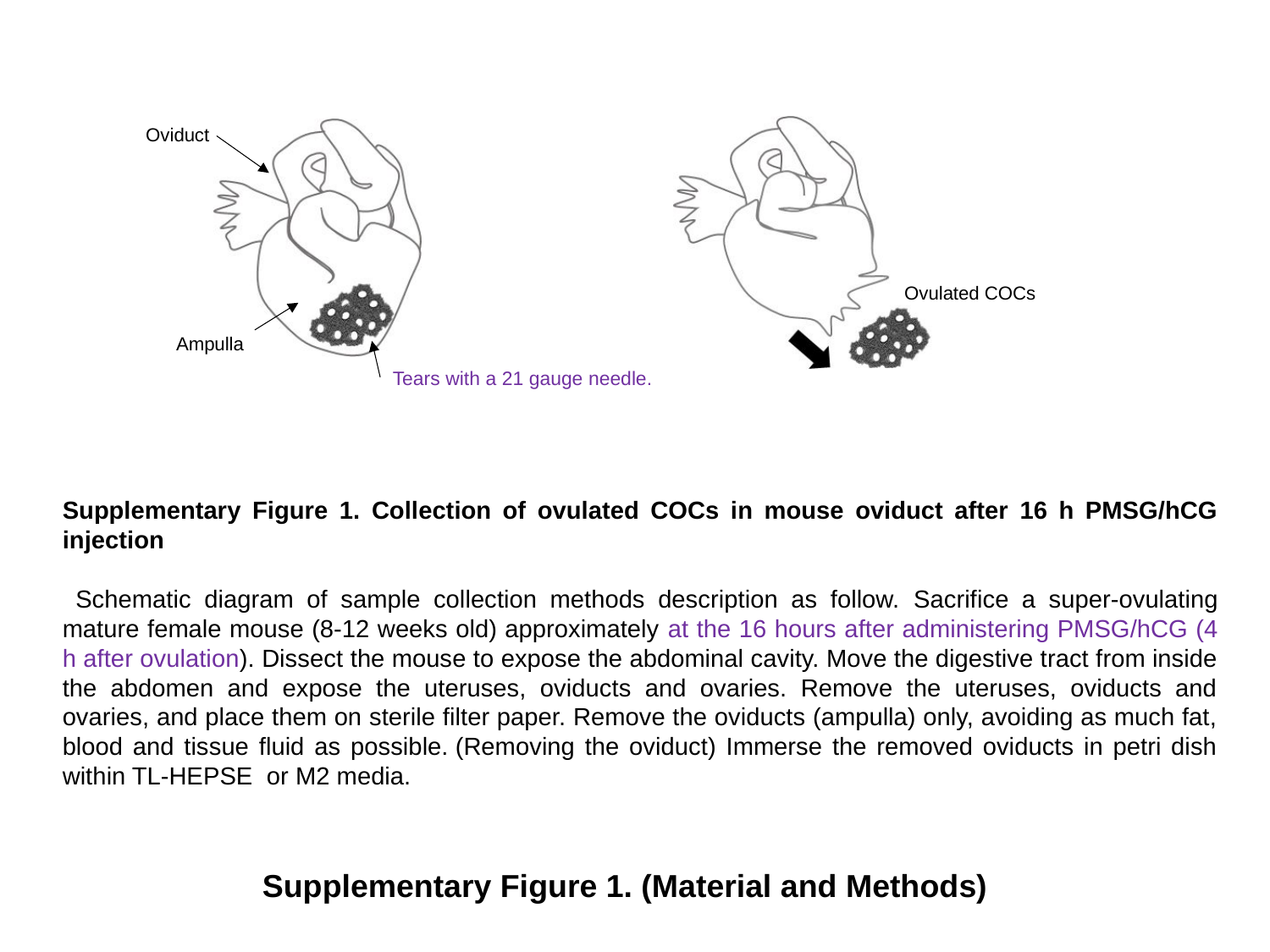

Oviduct
Ovulated COCs
Ampulla
Tears with a 21 gauge needle.
Supplementary Figure 1. Collection of ovulated COCs in mouse oviduct after 16 h PMSG/hCG injection
 Schematic diagram of sample collection methods description as follow. Sacrifice a super-ovulating mature female mouse (8-12 weeks old) approximately at the 16 hours after administering PMSG/hCG (4 h after ovulation). Dissect the mouse to expose the abdominal cavity. Move the digestive tract from inside the abdomen and expose the uteruses, oviducts and ovaries. Remove the uteruses, oviducts and ovaries, and place them on sterile filter paper. Remove the oviducts (ampulla) only, avoiding as much fat, blood and tissue fluid as possible. (Removing the oviduct) Immerse the removed oviducts in petri dish within TL-HEPSE or M2 media.
Supplementary Figure 1. (Material and Methods)
